# Editorial: Towards the Next Generation of Deep Brain Stimulation Therapies: Technological Advancements, Computational Methods, and New Targets

**DOI:** 10.3389/fnins.2021.737737

**Published:** 2021-08-19

**Authors:** Sabato Santaniello, George C. McConnell, John T. Gale, Rose T. Faghih, Caleb Kemere, Justin D. Hilliard, Martin Han

**Affiliations:** ^1^Biomedical Engineering Department and Institute for the Brain and Cognitive Sciences, University of Connecticut, Storrs, CT, United States; ^2^Department of Biomedical Engineering, Stevens Institute of Technology, Hoboken, NJ, United States; ^3^Gale Neurotechnologies Inc., Smoke Rise, GA, United States; ^4^Department of Electrical and Computer Engineering, University of Houston, Houston, TX, United States; ^5^Department of Electrical and Computer Engineering, Rice University, Houston, TX, United States; ^6^Department of Neurosurgery, University of Florida, Gainesville, FL, United States; ^7^Biomedical Engineering Department and Institute of Materials Science, University of Connecticut, Storrs, CT, United States

**Keywords:** movement disorders, psychiatric disorders, neurostimulation devices, basal ganglia, electrodermal activity, DBS lead, adaptive DBS, DBS surgery

Deep Brain Stimulation (DBS) has matured into a staple of modern therapeutics for movement disorders and is considered a promising tool toward the treatment of psychiatric conditions (Vedam-Mai et al., 2021). More importantly, DBS has been the engine propelling the development of a diverse ecosystem of technological innovations, ranging from surgical navigation systems that incorporate connectome data (Li et al., 2020) to algorithms that predict the therapeutic outcomes of brain stimulation (Gonzalez-Escamilla et al., 2019; Reich et al., 2019), and implantable neurostimulators that integrate chronic monitoring and real-time modulation of neural activity (Stanslaski et al., 2018; Topalovic et al., 2020).

With the wealth of technological advancements accrued in recent years, two key questions have rapidly gained interest: (1) How do stimulation targets and settings affect the therapeutic efficacy of DBS? and (2) How can we minimize the burden associated with DBS programming while maximizing the clinical efficacy? In this Research Topic we gathered original research studies from experts in the field who addressed these questions and provided cutting-edge solutions toward the next generation of DBS therapies.

Despite advancements in neuro-navigation and planning, the decision about precisely where to stimulate (i.e., *which electrode contact should be activated on a DBS array?*) remains challenging, in part because intraoperative feedback on lead placement often relies on expert interpretation of intra-operative multiunit recordings. To cope with this limitation, Ozturk et al. reported a double-blinded pilot study that showed the potential of novel intra-operative analyses based on local field potentials. The authors demonstrated that the analysis can be done online in the operating room and enhanced the therapeutic outcomes of DBS through improved target localization compared to current methods using multiunit recording. Li and McConnell reported interesting work in rats that mapped the heterogeneity of the electrophysiology in the substantia nigra (SNr) and the ventral tegmental area, which are promising new targets for DBS. The authors reported the existence of distinct electrophysiological features in these areas and showed how these features can help precisely target SNr subregions during DBS surgery.

To further enhance the therapeutic outcomes of DBS, Anderson et al. developed a novel directional DBS lead with thousands of microscale contacts. The new design dramatically increased the spatial resolution of stimulation steering and improved the selectivity in targeting small diameter fibers, which promises to significantly widen the window of therapy for DBS. Furthermore, Zheng et al. investigated the effects that can be induced on the therapeutic outcomes of DBS by changing the appearance order of the intervals between consecutive pulses. They showed that a random arrangement of inter-pulse intervals (IPI) can recruit more neurons to fire in synchrony following specific sub-sequences of pulses compared to gradual IPI, thus providing a paradigm to widen the neuronal recruitment in response to DBS.

A general consensus has been that the burden of DBS programming will be lowered by introducing closed-loop control algorithms. However, studies so far have mainly assessed the feasibility of closed-loop DBS over short periods (Little et al., 2013; Arlotti et al., 2018) and have been limited to control algorithms that lack sensitivity and specificity over long durations. Because symptom fluctuations are a hallmark of various movement disorders, occurring on multiple time scales, there is an unmet need for algorithms that can self-adapt as symptoms and biomarkers evolve with time. In this Research Topic, we presented work related to this need with respect to the fluctuations of pathological beta-band in Parkinson's disease (PD). Fleming, Dunn, et al. evaluated the resilience of traditional PI controllers against beta-band fluctuations, and Fleming, Orlowski, et al. proposed a novel, self-tuning controller that tracks beta-band fluctuations over time and adjusts the closed-loop DBS strategy accordingly. Also, Su et al. proposed a hierarchical control architecture, where the closed-loop DBS is based on an autoregressive (AR) model of the input-output relationship between DBS pulses and pathological beta-band oscillations. As the AR model is updated periodically through the day, the control strategy is automatically adjusted to efficiently cope with the daily fluctuations of beta-band oscillations. Finally, Cutsuridis expanded the model-based framework to develop DBS strategies for chronic memory loss treatment.

With regard to PD, DBS therapies have been traditionally focused on motor symptoms such as akinesia (Moro et al., 2010), even though cognitive symptoms are a significant contributor to the severe disability imposed by the disease, diminishing the individual's quality of life. An emerging trend suggests that DBS therapies should be used to satisfy multiple therapeutic goals simultaneously and address both motor and non-motor symptoms. This may necessitate the investigation of new targets, stimulation patterns, and feedback signals. In Bentley et al., authors capitalized on the positive cognitive outcomes of intermittent theta-burst stimulation (iTBS, a TMS paradigm) of the dorsolateral prefrontal cortex (DLPFC) and delivered DBS with iTBS pulse sequences to the globus pallidus of PD patients. They documented the effects of GPi iTBS vs. regular GPi DBS on the neuronal activity in the DLPFC, which is a center for PD cognitive symptoms, and reported evidence of the cognitive effects of DBS. Wickramasuriya et al., on the other hand, proposed the use of sympathetic arousal as a potential biomarker of non-motor symptoms. The authors specifically focused on neuropsychiatric symptoms and developed an innovative approach to efficiently decode psychological arousal from neural activity underlying skin conductance signal variations. Finally, Guo et al. investigated hybrid stimulation protocols to treat disorders of consciousness (DOC) and proposed a combination of deep stimulation and high-density transcranial direct current stimulation of the precuneus to rehabilitate DOC patients.

Altogether, these contributions showed that the next generation of DBS therapies will aim to expand the range of clinical applications and boost therapeutic outcomes through a rapid integration in the design process of wearable sensing modalities, electronic miniaturization, control methods, and electrophysiological exploration.

## Author Contributions

All authors contributed to edit the manuscript.

## Conflict of Interest

JG was employed by Gale Neurotechnologies Inc. The remaining authors declare that the research was conducted in the absence of any commercial or financial relationships that could be construed as a potential conflict of interest.

## Publisher's Note

All claims expressed in this article are solely those of the authors and do not necessarily represent those of their affiliated organizations, or those of the publisher, the editors and the reviewers. Any product that may be evaluated in this article, or claim that may be made by its manufacturer, is not guaranteed or endorsed by the publisher.
